# The unlikely combination: Anderson–Fabry disease and congenital dyserythropoietic anemia type II in a pediatric patient

**DOI:** 10.1002/ccr3.9354

**Published:** 2024-10-08

**Authors:** Yasmine Elsherif, Ismail A. Ibrahim, Omar Elsherif, Hana J. Abukhadijah

**Affiliations:** ^1^ Internal medicine American Hospital Dubai (AHD) Dubai UAE; ^2^ Faculty of Health Sciences Fenerbahce University Istanbul Turkey; ^3^ Tbilisi State Medical University Tbilisi Georgia; ^4^ Academic Health System Department Hamad Medical Corporation Doha Qatar

**Keywords:** Anderson‐Fabry disease, CDA type II, congenital dyserythropoietic anemia, co‐occurrence, genetic distinction, lysosomal disorder

## Abstract

**Key Clinical Message:**

Anderson‐Fabry disease, a rare X‐linked lysosomal disorder, and congenital dyserythropoietic anemia (CDA) Type II, an autosomal recessive condition, both have distinct inheritance patterns. Their co‐occurrence is extremely rare, never been reported before. Therefore, screening is crucial for early management, and families should seek genetic counseling for children showing unusual presentations.

**Abstract:**

Anderson‐Fabry disease (AFD) is a rare condition, characterized by a lysosomal storage disorder affecting lipid storage. It manifests in two forms: classic (early‐onset) and nonclassic (late‐onset). Conversely, congenital dyserythropoietic anemia (CDA) is a rare blood disorder caused by ineffective erythropoiesis, which results in the production of abnormal erythroblasts during the maturation of red blood cells, with CDA type II being the most frequent type. Both disorders have well‐understood pathophysiologies, yet they are genetically distinct. AFD is inherited in an X‐linked manner, whereas CDA type II follows an autosomal recessive pattern of inheritance. Although both AFD and CDA type II have been reported separately in the literature. The co‐existence for both AFD and CDA type II has not been reported. We describe a 10‐year‐old boy, with both which is believed to be the first documented case.

## 1 INTRODUCTION

Anderson–Fabry disease (AFD) is a lysosomal storage disorder with a defect in storing lipids leading to potentially life‐threatening conditions. In children, mainly the manifestation is of recurrent nonspecific gastroenteritis episodes.

Congenital dyserythropoietic anemia (CDAs) is a rare blood disorder characterized by ineffective erythropoiesis during erythrocyte maturation resulting in morphologically abnormal erythroblasts. This appears in 10%–50% of mature erythroblasts which is considered the hallmark feature of the CDA, CDA is divided into multiple types. CDA II type, known also as hereditary erythroblastic multinuclearity with positive acidified serum lysis test (HEMPAS), is the most common type of CDA. Generally, presents with anemia along with hepatosplenomegaly and cholecystolithiasis.^1^, ^2^, ^3^

AFD is an X‐linked disorder and CDA type II is an autosomal recessive pattern. The combined form of both entities of disorders isn't widely known. Since both CDAs and AFD are rare genetic disorders, the likelihood of an individual having both conditions simultaneously is exceedingly low. In this paper, we report a case of a child who was diagnosed with both disorders. To the best of our knowledge, it is the first case of AFD and CDA type II co‐existing in a 10‐year‐old boy.

## 2 CASE PRESENTATION

A 10‐year‐old boy presented to the pediatric hematology clinic with complaints of pallor and recurrent infections over the past few months. The onset of repeated viral and bacterial infections began in October 2022. Initially, symptomatic treatment measures were given, but was most recently, diagnosed with mycoplasma and received an appropriate antibiotic dose and duration of treatment. He was delivered through normal vaginal delivery (NVD), exclusively breastfed, and did not have any post‐natal concerns. His vaccination status were up to date. Regarding family history, he is the only child of a nonconsanguineous marriage. Notably, the mother and maternal grandmother have a history of unspecified anemia. There is no reported history of thalassemia, sickle cell disease, or leukemia/lymphoma in the family. The child and the parents are known vegetarian.

**FIGURE 1:**
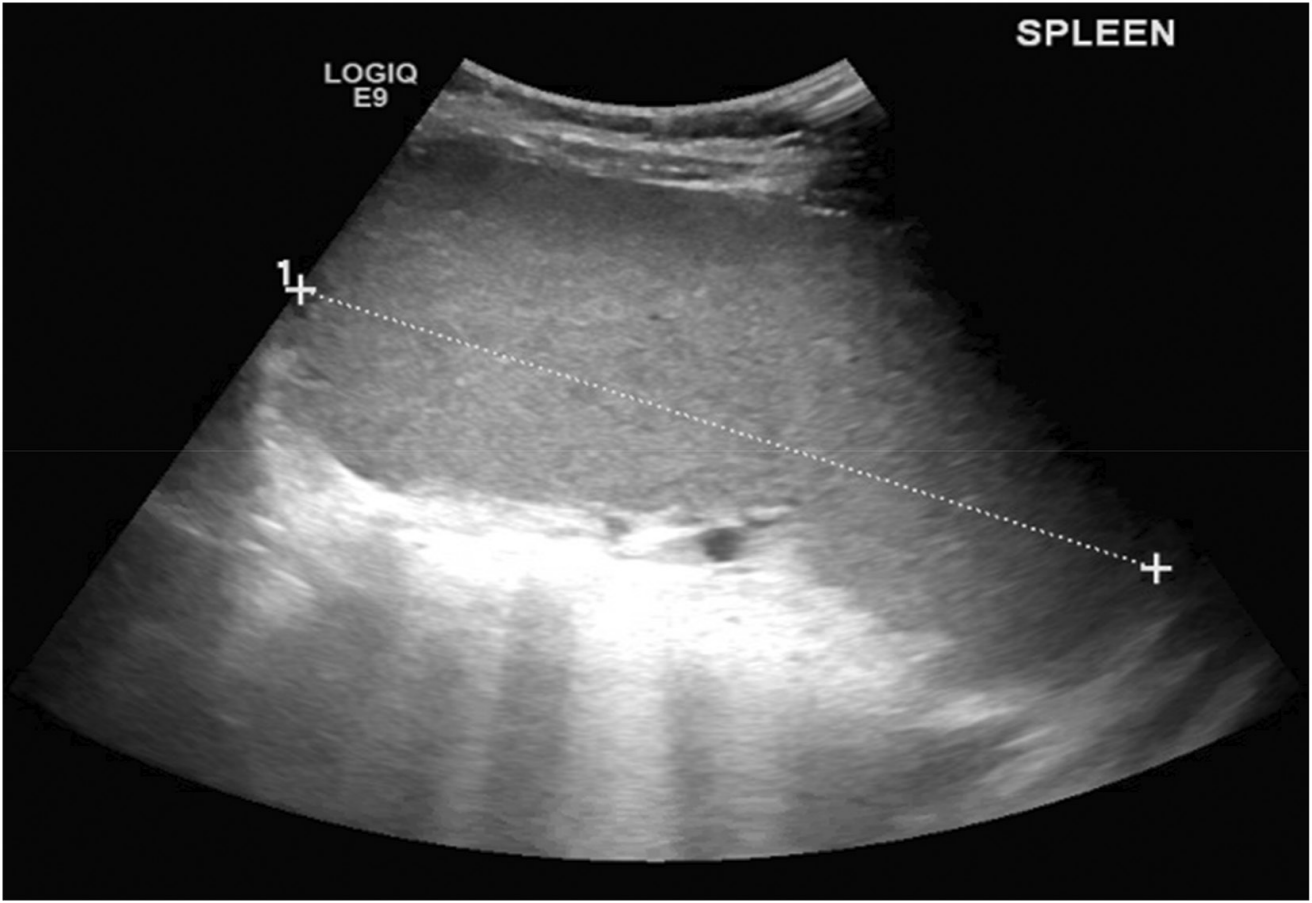
Image of a spleen on Ultrasound Abdomen. US showed spleen enlagrement with measured length of about 14.9 cm. (Refernce for Normal range for the patients age 6.5 ‐ 11 cm) and has a normal homogenous echotexture. No focal abnormality was displaed. No ascites seen.

**FIGURE 2:**
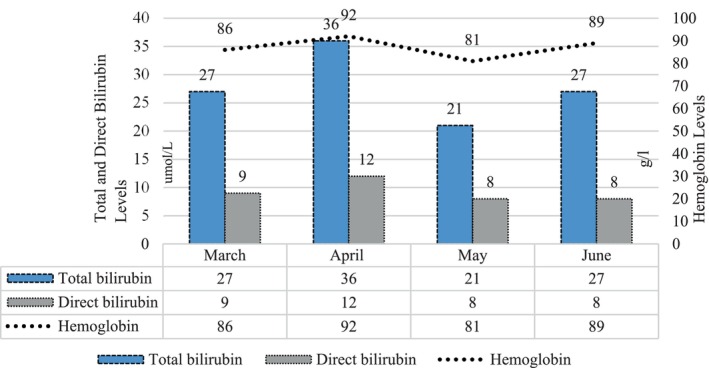
Illustrates the trends of bilirubin direct, bilirubin total, and hemoglobin levels.6. Obvious anemia even after Iron replacement therapy, and persistent unexplained increase of bilirubin.

Vitally stable, weight 35 kg and height 140 cm. On Physical Exam abdomen was soft, and none tender with a palpable spleen, 4 cm below the coastal margin. No hepatomegaly and no palpable mass were detected other systems yielded unremarkable. The Laboratory test results were the following Hemoglobin 84.0 g/L (112 to 145 g/L), Bilirubin Total 27.0 μmol/L (5.1–17 μmol/L), Bilirubin Direct 7.0 μmol/L (3.4–12.0 μmol/L), Ferritin 5.2 μg/L (5 and 20 μg/L). He was treated with Folic acid 5 mg orally once daily and 3 drops per Kg/day of Sucroma Iron (SiderAL® Gocce [0.7 mg/drop]) which was 2 mLs (30 drops) twice daily. TheHBelectrophoresis was normal. Ultrasound of the abdomen (US) demonstrated an enlarged spleen without a focal lesion (Figure 1). Osmotic fragility testing (OFT) was highly suggestive of spherocytosis. The dose of Sucroma Iron (SiderAL® Gocce [0.7 mg/drop]) was increased to 3 mLs twice daily. The pediatric gastroenterologist conducted a comprehensive evaluation of iron deficiency anemia, which involved an endoscopy. The endoscopic examination revealed normal findings (Figure 1), but labs showed anemia and abnormal bilirubin levels (Figure 2). The following figures were recorded for Total Bilirubin, Direct Bilirubin, and Hemoglobin across several months. In March, Total Bilirubin was 27 μmol/L, Direct Bilirubin was 9 μmol/L, and Hemoglobin was 86 g/L. In April, Total Bilirubin increased to 39 μmol/L, Direct Bilirubin was 12 μmol/L, and Hemoglobin was 92 g/L. In May, Total Bilirubin decreased to 21 μmol/L, Direct Bilirubin was 8 μmol/L, and Hemoglobin was 81 g/L in June, Total Bilirubin was 27 μmol/L, Direct Bilirubin was 8 μmol/L, and Hemoglobin was 89 g/L. The results of whole‐genome sequencing (WGS) indicated a diagnosis of both Fabry disease (FD) and CDA type II. SEC23B variant c.1385A > G p.(Tyr462Cys) amino acid change from Tyr to Cys at position 462. The zygosity is homozygotous. It is missense likely pathogenic (class 2) according to the recommendations of CENTOGENE and ACMG. The GLA variant c.770C > T p.(Ala257Val) amino acid change from Ala to Val at position 257. The zygosity is homizygotous. It is missense likely pathogenic (class 2) according to the recommendations of CENTOGENE and ACMG. Alpha‐ galactosidase leukocyte enzyme test was 0.89 nmol/h/mg which is consistent with FD. Referred to a pediatric neurologist, whom advised Agalsidase alfa 2 vials every 2 weeks for a total of 4 in a month. Yet to follow up with the insurance and the overall outcome of this treatment.

## 3 DISCUSSION

AFD is infrequent; estimated incidence is 1 in every 117,000 males.^2^ It is further classified into two forms: classic (early‐onset) and nonclassic (late‐onset). CDAs numbers vary according to the CD type. Divided into CD type I (CDAN1), II (CDAN2), and III (CDAN3); the latter type is the rarest form. The clinical relevance of this case is the combination of two vastly epidemiologically different inherited diseases. Therefore, making it challenging to predict the outcome in this young patient who is still free of organ involvement. A unique case similar to ours, in the sense that two different inherited diseases have been documented, is a case of a 3‐year‐old male with early onset of AFD found to have combined inborn disease of Phenylketonuria (PKU) and early‐onsite FD.^4^

The pathophysiology of both disorders is well known. AFD is caused by a mutation in the GLA gene (Agalsidasealfa, Alpha‐D‐galactosidase A). A gene that regulates the production and function of an enzyme called alpha‐galactosidase (α‐GAL) A. Absent or dysfunction of this enzyme will result in the build‐up of globotriaosylceramide (Gb3) and globotriaosylsphingosine (Lyso‐Gb3) especially Gb3 in any organs (e.g., heart, kidney, intestinal, and eyes disease).^5^, ^6^, ^7^, ^8^, ^9^

The symptoms of AFD can vary depending on the accumulation of Gb3 in different organs, including the heart, kidneys, brain, central nervous system, and skin. The patient primarily presented symptoms more consistent with CDA rather than FD, making the detection of FD incidental yet crucial for the patient's future health. Timely identification is essential as delayed diagnosis may lead to the onset of subsequent symptoms. The initial sign of classic AFD that would have been expected in our patient was paresthesia in the hands and feet, particularly triggered by fever and heat intolerance. This symptom is observed in approximately 59% of males and 41% of females. However, it frequently goes unnoticed as it presents episodically in the form of painful attacks known as Fabry pain crises.^10^ This element was absent in our case, further complicating the diagnosis. End‐Stage Renal Disease (ESRD), ventricular remodeling, and cerebrovascular disease could emerge years later after the onset of the initial symptoms. Furthermore, all symptoms mentioned above have been reported to occur in adolescence.^12^, ^13^, ^14^ Though these symptoms have not yet been observed in our patient, initiating treatment was crucial to prevent the listed complications. It is otherwise uncommon for juveniles to develop ESRD due to AFD. Typically presenting in their fifties. Therefore, our patient was strongly advised to follow up with both hematologist and neurologist for any emerging symptoms.

Mainly in children, the complaints are of recurrent nonspecific gastroenteritis episodes arising in roughly 90% of most patients.^11^ This was not the case in our patient, as he denied any history of gastroenteritis; his concerns were primarily related to an upper respiratory tract infection. A median age of onset in males of 5 and 9.5 years in females. Male tend to develop severe symptoms in comparison to females.^15^, ^16^, ^17^ Whereas CDA presents equally in both gender. CDA type II presents with a milder form of anemia along with signs of hepatosplenomegaly and cholecystolithiasis. This was evident in our case as the reason for his presentation, with him showing signs of anemia such as pallor, along with positive physical findings indicative of a hematological disease. The patient's vegetarian diet also initially pointed towards nutritional deficiencies as a possible cause of his anemia but did not address the frequent infections and the jaundice.

Differential diagnosis includes congenital spherocytic anemia, which is excluded by carrying out advanced tests such as OF and protein electrophoresis.

Both disorders are managed symptomatically, as there is currently no cure. Long‐term target therapy for AFD patients is enzyme replacement therapy (ERT) with or without chaperone therapy (e.g., migalastat) these are prophylactic measures recommended to be initiated as soon as possible to prevent and/or delay the complications. Agalsidasealfa (ReplagalTM) and, Agalsidase beta (FabrazymeTM) are recombinant human α‐GAL. A dose of 1.0 mg/kg approved for children and adolescents aged from 7 to 8 years and older.^18^ In our case, he was started on this regimen. The two forms of ERT are approved by the European Medicines Agency (EMA) and are available as an intravenous infusion (IV). Besides this annual clinical assessment is important, cardiologists encourage yearly electrocardiogram (EKG), echocardiogram, and renal function tests in 18‐year‐old males and older. In CDA, the confirmatory diagnostic tool is a bone marrow biopsy, and ham test.^19^ Laboratory findings is usually of elevated serum ferritin levels.

CDA modes of management are either surgical or nonsurgical interventions. The majority of patients need blood transfusions every 2–3 weeks and chelation therapy (e.g., deferoxamine, deferiprone) to avoid toxicity. Moreover, interferon alpha has shown evidence of improving hemoglobin levels.^20^, ^21^ Some may require bone marrow transplants along with cholecystectomy and splenectomy procedures, despite the latter being a controversial matter.^22^ Heimpel et al. conducted a retrospective study where they observed 48 patients with CDA type II for 35 years,^19^ describing their overall management. The outcome was that splenectomy is an optimal approach for patients with debilitating anemia, compromising their quality of life. Gene therapy is still an ongoing experimental treatment. The aim is to compensate for SEC23B deficiency in CDAII hematopoietic stem cells. The benefits of autologous hematopoietic stem cell transplant (HSCT) using genetically modified cells exceed those of allogeneic HSCT.^23^

## 4 CONCLUSION

CDA type II and AFD co‐occurring in the same patient is a very rare medical finding, this has never been reported. Both disorders are rare and genetically distinct on their own, but their co‐occurrence raises the possibility of intricate interactions and difficult diagnostic situations in clinical practice. This case emphasizes the importance of considering rare disease comorbidities and underscores the need for further investigation into potential genetic and pathophysiological links between these seemingly disparate conditions.

## AUTHOR CONTRIBUTIONS

**Yasmine Elsherif:** Conceptualization; data curation; resources; writing – original draft; writing – review and editing. **Ismail A. Ibrahim:** Resources; writing – original draft; writing – review and editing. **Omar Elsherif:** Resources; writing – review and editing. **Hana J. Abukhadijah:** writing – review and editing.

## CONFLICT OF INTEREST STATEMENT

The authors declare that they have no competing interests.

5

## CONSENT

Written informed consent was obtained from the patient to publish this report in accordance with the journal's patient consent policy.

## Data Availability

Data available on request from the authors.
